# Comparison of Milk Kefirs Obtained from Cow’s, Ewe’s and Goat’s Milk: Antioxidant Role of Microbial-Derived Exopolysaccharides

**DOI:** 10.3390/antiox13030335

**Published:** 2024-03-11

**Authors:** Sana M’hir, Lamia Ayed, Ilaria De Pasquale, Elisabetta Fanizza, Ali Zein Alabiden Tlais, Roberto Comparelli, Michela Verni, Rosanna Latronico, Marco Gobbetti, Raffaella Di Cagno, Pasquale Filannino

**Affiliations:** 1 Laboratory of Microbial Ecology and Technology (LETMi), National Institute of Applied Sciences and Technology (INSAT), University of Carthage, BP 676, Tunis 1080, Tunisia or sana.mhir@isbb.rnu.tn (S.M.); lamiaayed@yahoo.fr (L.A.); 2 Department of Animal Biotechnology, Higher Institute of Biotechnology of Beja, University of Jendouba, BP 382, Beja 9000, Tunisia; 3 CNR-IPCF, Istituto per i Processi Chimico-Fisici, S.S. Bari, c/o Dip. Chimica Via Orabona 4, 70126 Bari, Italy; i.depasquale@ba.ipcf.cnr.it (I.D.P.); elisabetta.fanizza@uniba.it (E.F.);; 4 Department of Chemistry, University of Bari Aldo Moro, Via Orabona 4, 70126 Bari, Italy; 5 National Interuniversity Consortium of Materials Science and Technology (INSTM), Bari Research Unit, 70126 Bari, Italy; 6 Faculty of Agricultural, Environmental and Food Sciences, Libera Università di Bolzano, 39100 Bolzano, Italy; 7 Department of Environmental Biology, “Sapienza” University of Rome, Piazzale Aldo Moro 5, 00185 Rome, Italy; michela.verni@uniroma1.it; 8 Department of Soil, Plant and Food Science, University of Bari Aldo Moro, 70126 Bari, Italy; rosanna.latronico@uniba.it

**Keywords:** monosaccharides, polysaccharides, antiradical, reducing power, ABTS, FRAP, DPPH, fermentation, metabolism, substrate

## Abstract

Different types of milk are used in the production of milk kefir, but little information is available on the release of potentially antioxidant exopolysaccharides (EPS). The aim of this study was to investigate whether the microbial dynamics and EPS release are dependent on the milk substrate. In our study, the inoculated microbial consortium was driven differently by each type of milk (cow, ewe, and goat). This was evident in the sugar consumption, organic acid production, free amino release, and EPS production. The amount and the composition of the secreted EPS varied depending on the milk type, with implications for the structure and functional properties of the EPS. The low EPS yield in ewe’s milk was associated with a higher lactic acid production and thus with the use of carbon sources oriented towards energy production. Depending on the milk used as substrate, the EPS showed different monosaccharide and FT-IR profiles, microstructures, and surface morphologies. These differences affected the antiradical properties and reducing power of the EPS. In particular, EPS extracted from cow’s milk had a higher antioxidant activity than other milk types, and the antioxidant activity was concentration dependent.

## 1. Introduction

Milk kefir (MK) is a popular fermented beverage that has gained widespread popularity across various geographical regions, and has several health benefits [1]. MK is obtained by inoculating milk with kefir grains or microbial cultures, which consist of different types of bacteria and yeasts. The nutritional and functional value of MK stems from its unique composition, including proteins, peptides, amino acids, vitamins, minerals, and other bioactive molecules of microbial origin [1]. The health-promoting properties of MK are closely linked to microbial dynamics, which are particularly influenced by the type of milk used, and these variations can present challenges in establishing standardized MK production methods. While milk naturally contains peptides with biological activities, the microbial proteolytic activity during milk kefir (MK) fermentation significantly enhances the peptide content. These peptides may exert a number of beneficial effects, including antimicrobial, antioxidant, angiotensin-converting enzyme (ACE)-inhibitory, immunomodulatory, and antithrombotic effects [2,3,4,5,6]. Due to the high protein content of dairy products, the role of microbial enzymes in the release of free amino acids and peptides potentially responsible for antioxidant activity has been extensively studied. In contrast, less attention has been paid to the synthesis and properties of potentially antioxidant exopolysaccharides (EPS). During MK fermentation, microbial cells are carried by grains consisting of kefiran, a branched water-soluble EPS produced by some lactobacilli. Theoretically, the glucose–galactose ratio in kefiran should mirror that of the lactose disaccharide, but variations ranging from 1:0.4 to 1:1.88 have been reported [7]. Kefiran has many beneficial properties, including antioxidant, anti-inflammatory, and immunomodulatory activity [8]. Kefiran has been isolated from various lactobacilli found in kefir (e.g., *Levilactobacillus brevis*, *Lactobacillus kefiranofaciens*, *Lactiplantibacillus pentosus*, *Lactobacillus helveticus*), with varying types of linkages, branching, and residues of glucose, galactose, mannose, arabinose, and rhamnose contributing to a diverse range of functional capabilities [8]. A notable example that extends this understanding is the EPS produced by *Lacticaseibacillus paracasei* strains isolated from kefir grains. Research has shown that the properties of these EPS vary with fermentation temperature, which not only affects their rheological properties in fermented dairy products, but also highlights their diverse functional capabilities in general [9]. In addition to lactobacilli, other polysaccharide-producing species have also been isolated from MK (e.g., *Streptococcus thermophilus*, *Leuconostoc mesenteroides*) [10,11]. As well as being associated with grains, EPS can be released into milk and accumulate in cell-free form [12]. Studies have demonstrated that MK-derived EPS can act as an effective antioxidant. The antioxidant potential of EPS has generally been linked to their structural properties, including the type of monosaccharides, glycosidic linkages, molecular weight, and the presence of certain groups such as carbonyl, sulphate, amino, and carboxyl. This relationship between structure and antioxidant activity is mainly due to the ability of different structural elements to influence the hydrogen-donating capacity of EPS [13]. For example, the composition of monosaccharides plays an important role in determining the ability of EPS to scavenge free radicals. Different monosaccharides confer different levels of antioxidant activity and have a direct impact on the effectiveness of EPS as antioxidants. Specifically, glucose, mannose, and galactose have been identified as key contributors to the substantial enhancement of the antioxidant capacity of EPS [14]. The type of glycosidic linkages and branching patterns within the EPS structure are also important. The antioxidant activity is significantly influenced by the flexibility imparted by α-1,2 and α-1,6 glycosidic bonds, in contrast to the comparatively more rigid β-1,3 and β-1,4 bonds [13]. These characteristics affect the stability and reactivity of EPS and influence their ability to neutralize oxidative agents. Functional groups in the monosaccharide units, such as hydroxyl groups (-COOH, C=O, and -O-), contribute significantly by donating protons to unpaired electrons of radicals and thus play a key role in the antioxidant mechanism of EPS [15,16,17,18,19]. 

Cow’s milk is the most common type of milk used in the industrial production of MK, but other types of milk (e.g., ewe, goat, camel, mare, and buffalo milk) can also be used. There is a growing demand for goat’s milk products due to the small diameter of the fat globules and their high digestibility [20]. In addition, goat’s milk contains higher levels of certain amino acids compared to cow’s milk proteins [21]. The nutritional and functional properties of goat’s milk proteins are remarkable, offering higher digestibility, buffering capacity, and alkalinity than cow’s milk. These properties make goat’s milk particularly suitable for people who are sensitive to cow’s milk or have lactose intolerance [21]. In addition, ewe’s milk is an important source of bioactive compounds with health-promoting functions. It is rich in fatty acids, immunoglobulins, proteins, hormones, vitamins, and minerals. Ewe’s milk is also a source of peptides with antibacterial, antiviral, and anti-inflammatory properties, and even has anti-cancer properties [22]. Thus, the nutritional and functional features of MK depend not only on the starter grains but also on the type of milk used [23,24,25]. Variations in EPS production may also occur, especially considering that EPS-producing bacteria are only a fraction of the microorganisms involved in MK fermentation, along with other lactic acid bacteria, acetic acid bacteria, and yeasts [23,26]. Many reports have shown that the amount and properties of EPS depend on the microorganisms involved in the fermentation process and the composition of the culture media, suggesting that the unique composition of each milk may result in different properties of EPS in MK [25]. 

Therefore, the aim of this study was to verify whether different milk types (cow, ewe, or goat) could affect the characteristics of MK, and especially the structure of EPS secreted and accumulated during fermentation. While prior studies have extensively examined EPS during milk production, they have largely overlooked the impact of the same starter culture across various milk types and conditions [12,27]. In our study, we monitored fermentation in terms of sugar consumption and organic acid production (as indicators of primary metabolism) and the release of free amino acids (as indicators of proteolysis). Then, the EPS have been characterized in terms of compositional and antioxidant features, in order to highlight the specific characteristics of each production.

## 2. Materials and Methods

### 2.1. Milk Kefir Fermentation

Kefir grains were provided by LETMi laboratory (Tunis, Tunisia) and consisted of a symbiotic consortium of lactic acid bacteria (*Leuconostoc* spp., *Lactococcus* spp., and lactobacilli) and yeasts (*Saccharomyces* spp. and *Zygosaccharomyces* spp.), as previously characterized [28]. Grains were routinely propagated in pasteurized cow’s milk at 25 °C. To carry out the fermentation trials with different types of milk, kefir grains were harvested by filtration after an overnight culture and inoculated (5%, *v v*^−1^) into 300 mL of pasteurized cow (CM), ewe (EM) and goat milk (GM). Milk was provided by a farm in Béja, northwest Tunisia, in March and April 2021. Milk was fermented in 500 mL Erlenmeyer flasks at 25 °C for 24 h. These fermentation parameters were adopted to support the optimal growth and metabolic activity of the kefir grain microbiota, resulting in the desired taste, texture, and nutritional properties of the kefir [12,29,30]. Unfermented (CM, EM, GM) and fermented (FCM, FEM, FGM) samples of cow’s, ewe’s, and goat’s milk were characterized as described below.

### 2.2. Microbiological Characterization

Presumptive mesophilic lactobacilli and cocci were enumerated in MRS agar (Oxoid, Basingstoke, Hampshire, UK) supplemented with cycloheximide (0.1 g L^−1^) and on M17 agar (Oxoid), respectively, under anaerobic conditions at 25 °C for 48 h. Yeasts were counted at 25 °C for 48 h by using Yeast Extract Peptone Dextrose (YPD) agar (Sigma-Aldrich, Milan, Italy) supplemented with chloramphenicol (0.1 g L^−1^).

### 2.3. Physicochemical and Rheological Characterization

The pH value was measured by direct insertion of a FoodTrode Electrode (Hamilton, Bonaduz, Switzerland). Total titratable acidity (TTA) was measured on 10 mL of milk homogenized with 90 mL of distilled water and expressed as the amount (mL) of 0.1 M NaOH needed to achieve a pH of 8.3. Color was measured using a Minolta CR-10 Camera. L*a*b* color space analysis method was used, where L* represents lightness (white–black) and a* and b* the chromaticity co-ordinates (red–green and yellow–blue, respectively). Color difference, ΔE*_ab_, was calculated as follows:

$$\Delta {E^{*}}_{\mathrm{ab}}=\sqrt{\Delta a^{2}+\Delta b^{2}+\Delta L^{2}}$$

Apparent viscosity was measured on conditioned (25 °C for 30 min) samples (35 mL) through the sine wave vibro-viscometer A&D SV-10 (A&D Company Ltd., Tokyo, Japan).

### 2.4. Sugars, Ethanol, and Organic Acids Quantification

Concentrations of sugars and ethanol were determined by HPLC analysis using a Spherisorb column (Waters, Millford, CT, USA) and a Perkin Elmer 200a refractive index detector [31]. Lactic and acetic acids were determined by HPLC analysis equipped with an Aminex HPX-87H column (ion exclusion, Biorad, Hercules, CA, USA) and a UV detector operating at 210 nm [31]. Sugars, ethanol, and organic acid standards were purchased from Sigma-Aldrich.

### 2.5. Total and Individual Free Amino Acids (FAAs)

Total and individual FAAs were determined using a Biochrom 30+ series Amino Acid Analyzer (Biochrom Ltd., Cambridge, UK) with a Li-cation-exchange column (20 by 0.46 cm inner diameter), as previously described by Verni et al. [32].

### 2.6. Purification of EPS 

EPS were purified by using the method described by Chen et al. [12]. Milk samples were heated for 10 min at 100 °C to inhibit enzyme activity after removing the kefir grains through qualitative cellulose-based filter paper, and subsequently centrifuging at 8000× *g* for 15 min. EPS were precipitated by the addition of 3 × the volume of chilled 95% ethanol (−20 °C, 12 h) to the supernatant fluid. After holding at 4 °C for 2 h, the samples were centrifuged at 8000× *g* for 15 min. The polysaccharide sediment was then re-dissolved in 20 mL distilled water and then mixed with 4 mL 10% trichloroacetic acid. The resulting mixture was incubated under stirring conditions (30 min at 120 rpm and 20 °C) in a shaking incubator. During agitation, proteins in the polysaccharide extracts were precipitated and discarded after centrifugation at 10,000× *g* for 15 min. The resulting supernatant was adjusted to pH 7.0 and the EPS were precipitated by adding an equal volume of chilled ethanol at 4 °C for 12 h. The solid precipitate was dissolved in double distilled water and the small neutral sugars were removed by dialysis through SpectraPor^®^ (Repligen, MA, USA) regenerated cellulose membrane (cut-off: 8000 Da) at 4 °C for 12 h, against three changes of distilled water. The residue inside the dialysis tube was freeze-dried and quantified by weight. The amount of EPS was expressed as the weight of EPS (in grams) produced per liter of milk culture.

### 2.7. DPPH^·^ Radical Scavenging Activity

The DPPH^·^ (2,2-diphenyl-1-picrylhydrazyl) radical scavenging capacity of the purified EPS was assessed according to the method described by Bouallegue et al. [33]. Different concentrations of purified EPS (0.1, 0.5, 1.0, 1.5, and 2.0 mg mL^−1^) were dissolved in water to prepare solutions for EPS testing. Five hundred microliters of variously diluted EPS were mixed with 375 μL of ethanol at 99.5% and 125 μL of DPPH^·^ (0.02%) dissolved in ethanol. After incubation in the dark at 25 °C for 30 min, the absorbance was measured spectrophotometrically at 517 nm. A mixture of DPPH^·^ and ethanol was used as control. The scavenging activity was expressed as follows: DPPH^·^ scavenging activity (%) = [(blank absorbance − sample absorbance)/blank absorbance] × 100.

### 2.8. Ferric Reducing Antioxidant Power (FRAP)

The ferric reducing power of EPS was examined using the method described by Bouallegue et al. [33], with some modifications. Five hundred microliters of variously diluted EPS (0.1–2 mg mL^−1^) was mixed with 1.25 mL of phosphate buffer (0.2 M, pH 6.6) and 1.25 mL of potassium ferricyanide 1% (*w v*^−1^). After incubation (50 °C, 20 min), the mixture was treated with 1.25 mL of trichloroacetic acid (10% *w v*^−1^) and centrifuged for 10 min at 3000× *g*. The supernatant (1.25 mL) was thoroughly mixed with ultra-pure water (1.25 mL) and 0.25 mL of a 0.1% (*w v*^−1^) ferric chloride solution. The absorbance was measured at 700 nm after incubation (25 °C, 10 min). FRAP value was expressed as mmol Fe^2+^ per ml.

### 2.9. ABTS^·+^ Radical Scavenging Activity

The ABTS working solution II was prepared following the method of Groth et al. [34]. Afterwards, twenty microliters of variously diluted EPS (0.1–2 mg mL^−1^) was mixed with 200 µL of ABTS working solution II. The absorbance was measured at 730 nm after 6 min at 30 °C by using a microplate reader (Biorad, Hercules, CA, USA). Radical scavenging activity was expressed as μmol Trolox equivalents (TE) per mL.

### 2.10. Fourier Transform Infrared Spectroscopy (FT-IR)

The FT-IR characterization was carried out by using a Varian 670 FT-IR spectrometer (Varian, CA, USA) equipped with a diamond ATR accessory of 2 mm and a DTGS (deuterated tryglicine sulfate) detector. The powder of each sample was placed on the internal reflection element and the spectra were recorded in the range 4000–400 cm^−1^, acquiring 16 scans with a nominal resolution of 1 cm^−1^.

### 2.11. Monosaccharide Composition of EPS

To hydrolyze the EPS, 15% (*v v*^−1^) perchloric acid (70%) was added, and the samples were heated at 80 °C for 1 h. To precipitate perchlorate, 250 µL of 5 M KOH was added. Precipitated potassium perchlorate was removed by centrifugation (12,000× *g*, 5 min, 4 °C), and the supernatant was used for the analysis of monosaccharides [35]. The monosaccharide composition was analyzed by HPLC using a Spherisorb column (Waters) as described above. For peak identification, arabinose, fructose, glucose, xylose, galactose, and rhamnose were used as external standards (Sigma-Aldrich).

### 2.12. Scanning Electron Microscopy (SEM)

Scanning electron microscopy (SEM) was performed by using a Zeiss Sigma microscope (Carl Zeiss Co., Oberkochen, Germany) operating in the range of 3 kV and equipped with an in-lens secondary electron detector and an INCA Energy Dispersive Spectroscopy (EDS) detector (Oxford Instruments, Abingdon, UK). EPS samples were used in the freeze-dried form. Subsequently, samples were mounted onto stainless steel sample holders by using double-sided carbon tape. A thin layer of Au (10 nm) was deposited via sputtering immediately prior to introducing the samples into the vacuum chamber of the SEM. This allowed for the mitigation of the charging effect caused by the electron beam accumulation. 

### 2.13. Statistical Analysis

For each condition, samples obtained from three independent experiments were analyzed in triplicate. Data were subjected to one-way ANOVA; pairwise comparison of treatment means was achieved by Tukey’s procedure at *p* < 0.05, using the statistical software Statistica for Windows (Statistica 7.0 for Windows, Statsoft Inc., Tulsa, OK, USA).

## 3. Results and Discussion

### 3.1. Microbiological, Physicochemical, and Rheological Characterization of Milk Kefirs Obtained from Cow, Ewe, and Goat Milk

After fermentation, the cell density of lactic acid bacteria and yeasts was in the range between ca. 9 and 10 Log CFU mL^−1^, with no statistically significant differences among the milk kefir samples (*p* > 0.05) [36]. The values of the physicochemical parameters measured in the unfermented (CM, EM, GM) and fermented (FCM, FEM, FGM) samples are summarized in Table 1. After 24 h of fermentation, the highest (*p* < 0.05) values of total titratable acidity (TTA) were found in the FEM, accordingly with the lowest (*p* < 0.05) pH values. Acidification in the FCM and FGM was less (*p* < 0.05) intense. Öner et al. [37] reported more pronounced acidification in ewe MK compared to cow’s and goat’s milk. The different degrees of acidification suggested that fermentation followed different microbial dynamics depending on the intrinsic differences in the milk’s initial acidity, nutrient composition, and the unique microbial interactions fostered by each milk type. Ewe’s milk, with its richer nutrient profile and buffering capacity, is likely to support a more vigorous fermentation process, resulting in greater acid production. Microbial dynamics, including the growth and metabolic activities of specific bacterial and yeast strains present in the kefir grains, are crucial factors that vary with the milk substrate [38]. 

Viscosity appeared to be influenced mainly by fermentation, with significantly higher values (*p* < 0.05), rather than by milk type (Table 1). Acidification is the primary driver of stable gel formation in milk [39]. However, milk composition, especially protein and fat content, can also contribute to higher viscosity [40]. Additionally, microbial metabolism can cause other events that influence viscosity. Several reports have stated that microbial-derived EPS can affect the firmness and texture of fermented milks [41,42]. EPS, due to their ability to bind water and to interact with proteins, may enhance the viscosity and the pseudoplastic behavior of fermented dairy products. It is clear that the production of EPS depends on the type of microorganisms involved and the conditions under which they grow [43,44]. In addition, the structure of the milk proteins can be weakened by proteolytic degradation during fermentation, which affects the viscosity of the kefir [45]. According to Saygili et al. [46], the rheological properties of MK varied depending on the type of milk and the incubation temperature. In particular, they reported lower apparent viscosities in MK obtained from ewe’s and goat’s milk compared to cow’s milk, and the increase in temperature significantly affected the fluidity of the kefir. These findings emphasize the impact of fermentation conditions and milk type on the rheological behavior of kefir, adding another layer of complexity to the factors influencing viscosity in fermented milk products.

The color was poorly modified by fermentation, with the exception of cow’s milk, for which fermentation resulted in a significant (*p* < 0.05) increase in L* and b* values (Table 1).

### 3.2. Microbial Metabolites

As expected, lactose was consumed (34–50%) during fermentation, resulting in the release of lactic acid (14.06–21.80 g L^−1^) and a small amount of acetic acid (0.29–0.43 g L^−1^) (Table 2). The highest (*p* < 0.05) lactic acid release was found in the FEM, in agreement with the TTA and pH values. Small amounts of glucose, galactose, and xylitol were released during fermentation (Table 2). Overall, an uneven profile is observed between the three types of milk. This suggests a diversified use of carbon sources. Guangsen et al. [38] monitored the microbiota during the fermentation of kefir from cow, ewe, and goat milk. Apart from a few dominant and homogeneous lactic acid bacteria species, the abundance of other sub-dominant species varied according to milk type, with *Lentilactobacillus parakefiri* and *Lactiplantibacillus plantarum* being most abundant in goat and cow MK, and *Lactococcus* spp. in ewe MK. Factors such as the protein, fat, and lactose content vary significantly between ewe’s, goat’s, and cow’s milk and influence the microbial growth and metabolism. For example, ewe’s milk, with its higher fat and protein content, supports the proliferation and activity of certain bacteria such as *Lactobacillus kefiranofaciens*, *Acetobacter syzygi*, *Lactococcus lactis,* and *Leuconostoc pseudomesenteroides* more effectively than goat’s or cow’s milk. These dynamics result in a distinct metabolic profile between different milk types, even when using the same starter. The abundance of the aforementioned bacterial species in ewe’s milk contributes to the production of a number of volatile compounds, including acetic acid and phenethyl alcohol, which are found in higher concentrations in ewe’s MK. They impact different flavors and odors, along with others such as 2-butanone, acetoin, and 2-propanone, which are unique to ewe’s MK [38].

### 3.3. FAA Profiles

**Figure 1:**
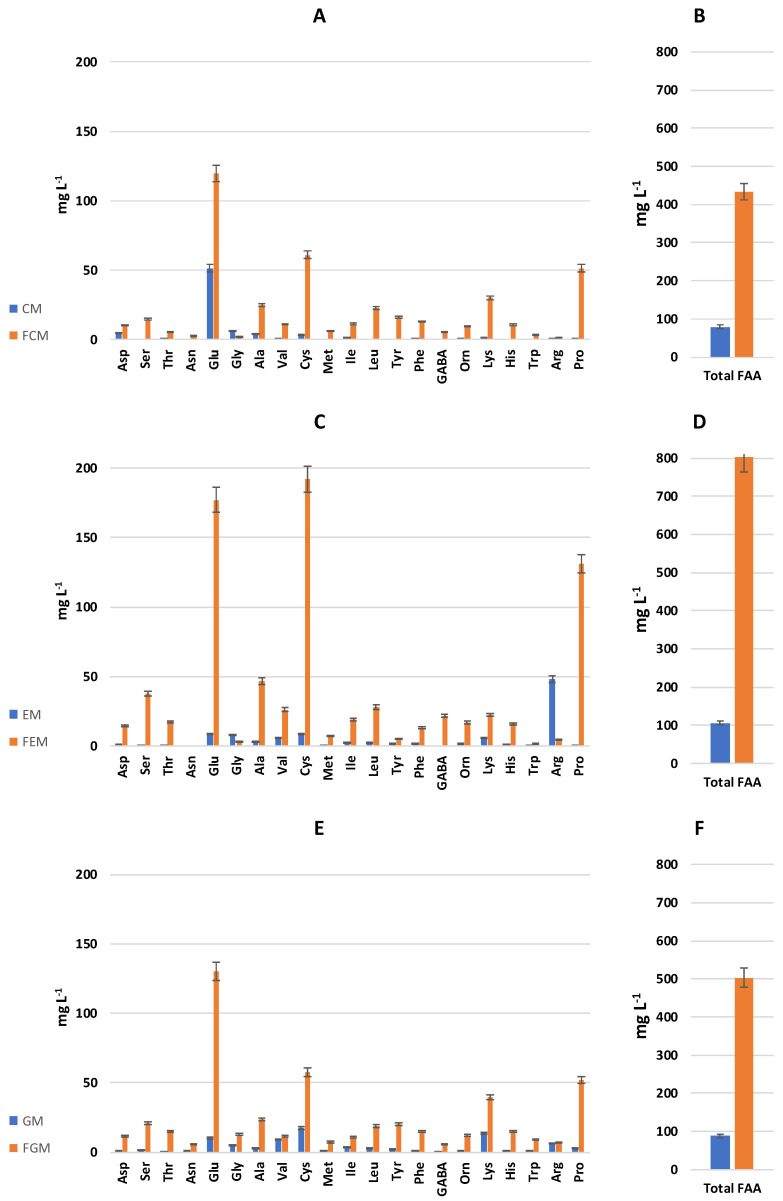
Concentrations (mg L^−1^) of free amino acids (FAAs). FAAs were determined in unfermented samples (blue bars) and fermented (25 °C for 24 h, orange bars) samples of cow’s (**A**,**B**), ewe’s (**C**,**D**), and goat’s milk (**E**,**F**). GABA stands for γ-aminobutyric acid.

As a consequence of the proteolysis performed by microorganisms on the native proteins of milk, the fermentation led to a strong (*p* < 0.05) increase in free amino acid release (447–656%) in the cow, ewe, and goat milk (Figure 1). The highest (*p* < 0.05) increment was observed in the FEM (Figure 1). The amount of FAAs represents an index of the degree of proteolysis and is related to the increase in protein digestibility in foods [47]. 

Compared to the unfermented milk samples, higher concentrations were found for most amino acids, with median increases of circa 16-, 10-, and 8-fold in the FCM, FEM, and FGM, respectively. In all the fermented milks, the highest levels were found for Glu, Cys, and Pro (Figure 1), with the FEM having the highest concentrations (177.04, 192.18, and 131.09 mg L^−1^, respectively). In line with these observations, a study by Gamba et al. [48] demonstrated that microbial proteolysis significantly elevates free amino acids, including glutamic acid, in both cow’s milk and soy milk kefir. In addition, a strong increase in Lys was also found in the FGM, with a final concentration of 39.65 mg L^−1^ (Figure 1). Besides the native proteins present in the substrate, the release and accumulation of FAAs depend on the activity of microbial proteolytic enzymes, mainly peptidases, and the extracellular translocation of amino acids when they exceed metabolic requirements, as discussed by Collar et al. [49]. The specificity of the peptidase cleavage sites determines the type of amino acids released. Additional reactions can occur that use amino acids as substrates, such as the deamination of glutamine and the transamination of other amino acids with alpha-ketoglutarate to produce glutamic acid [50]. This highlights the critical role of microbial proteolysis in amino acid enrichment.

Moreover, the bioavailability and digestibility of these amino acids in kefir are enhanced by proteolytic activities, as detailed by Ziarno et al. [51], contributing significantly to the nutritional value and digestibility of kefir. 

### 3.4. EPS Purification and Quantification 

The amount of EPS produced during milk kefir fermentation is largely affected by microbial cultures and process conditions. Under the conditions of our study, higher (*p* < 0.05) amounts of EPS were determined in the FCM and FGM (360 ± 24 and 380 ± 36 mg L^−1^) with respect to the FEM (300 ± 15 mg L^−1^). These differences were likely caused by different microbial dynamics driven by the milk composition. For example, the low EPS yield in ewe’s milk was associated with a higher lactic acid production (Table 2) and thus with the use of carbon sources oriented towards energy production in the context of lactic acid fermentation. The EPS yields were also compatible with the slight increase in viscosity in the fermented samples (Table 1). Other authors have reported EPS yields ranging from 57.2 to 223.3 mg L^−1^ in MK fermented with kefir grains [12,52], while yields reported for fermentations with pure cultures are much higher, exceeding 1000 mg L^−1^ [53]. 

### 3.5. Antioxidant Properties of EPS

**Figure 2:**
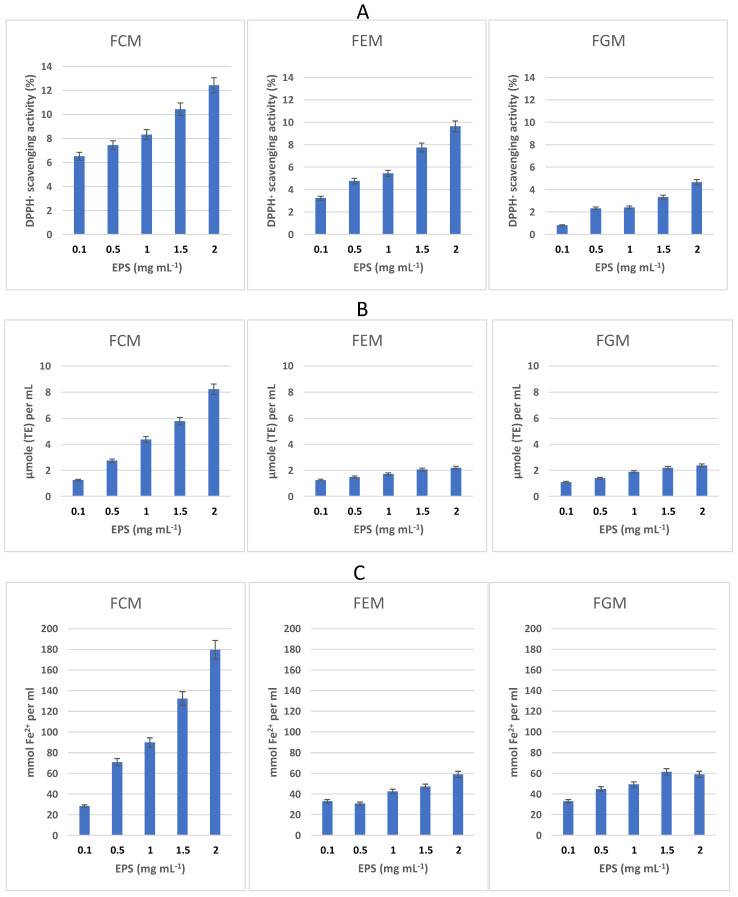
Antioxidant properties of EPS. DPPH^·^ radical scavenging activity (**A**), ABTS^·+^ radical scavenging activity (**B**), and ferric reducing antioxidant power (**C**) of EPS (0.1–2 mg mL^−1^) purified from cow, ewe, and goat milk fermented at 25 °C for 24 h (FCM, FEM, FGM).

As shown by the DPPH^·^ and ABTS^·+^ radical scavenging assays, EPS exerted significant antiradical activity in a dose-dependent manner (from 0.1 to 2 mg mL^−1^) (Figure 2A,B). Overall, the highest (*p* < 0.05) activity was observed in EPS from the FCM (Figure 2A,B). Monosaccharides with hydroxyl groups as well as other functional groups (e.g., amino groups) of EPS may donate a significant amount of hydrogen or electrons when interacting with free radicals during scavenging processes [19]. 

The reducing power of EPS was assessed by their ability to reduce ferric ions into ferrous ions in the FRAP assay (Figure 2C). Monosaccharides have an electron-donating ability due to the presence of reducing sugars that can donate an electron to the reactant. Certain functional groups present in the sugar molecule, such as sulfate, acetyl, carboxyl, hydroxyl, carbonyl, sulfhydryl, and thioether, can facilitate the metal binding process by forming charge transfer complexes with electron acceptors. These groups can also inactivate highly reactive molecules through a single electron transfer mechanism and activate the hydrogen of the anomeric carbon [19]. As reported in Figure 2C, the FRAP in the EPS was confirmed to be dose-dependent, and the most intense (*p* < 0.05) activity was found in the FCM EPS.

The differences in the antioxidant activity of the EPS obtained from the three types of milk can only be attributed to structural differences, including the chain conformation, monosaccharide content, and configuration of the glycosidic linkage, among others, and is probably not due to one single factor but to the interaction of several factors [54].

### 3.6. Monosaccharide Composition of EPS

Differences in the monosaccharide composition were found after the hydrolysis of purified EPS from the FCM, FGM, and FEM. Overall, the presence of different sugar moieties implies that the EPS produced during MK fermentation were heteropolysaccharides. EPS from FCM were composed of glucose, galactose, and rhamnose in an approximate ratio of 0.08:1:0.05. EPSs from FEM and FGM were composed of trehalose, glucose, galactose, and rhamnose in the approximate molar ratio of 0.07:0.09:1:0.05 and 0.08:0.09:1:0.09, respectively. These compositions differed from the EPS composed of glucose and galactose formed in kefir grains, as described by Chen et al. [12] and Blandón et al. [55]. *Lacticaseibacillus paracasei* isolated from kefir grains has been reported to produce EPS composed of glucose and mannose [56]. The components of kefiran reported by Koçak et al. [57], consisting of sucrose, glucose, galactose, arabinose, xylose, and ribose, also diverge from our findings. This variation in the monosaccharide profile is likely due to differences in milk composition, which affect microbial gene expression and dynamics during fermentation, leading to variations in the structure and antioxidant activity of EPS. It is not surprising to observe variations in the sugar composition of EPS, even when the same kefir grains are used as an inoculum. In fact, some authors have already demonstrated that the properties of EPS synthesized from pure cultures may differ from those synthesized in cocultures in terms of composition, thermodynamic properties, and surface morphology [58,59]. This highlights the importance of microbial interactions in complex microbial consortia and how the pressure exerted by the growth substrate alters these interactions, ultimately affecting EPS synthesis [60].

### 3.7. FT-IR 

**Figure 3:**
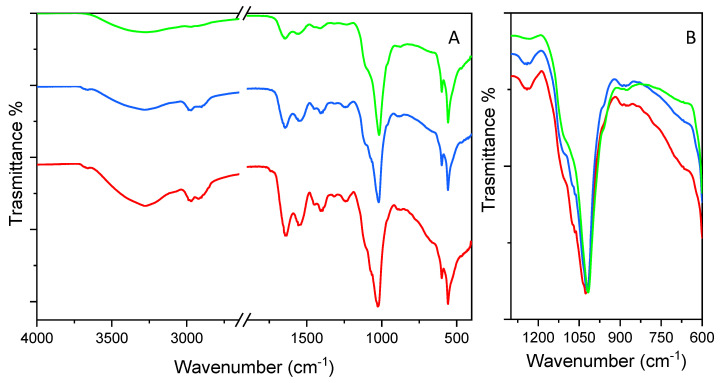
FT-IR spectra in ATR mode (**A**) in the 4000–400 cm^−1^ spectral range (break between 2600 and 1900 cm^−1^) and (**B**) in the 1300–600 cm^−1^ spectra range. The spectra of EPS from FCM (red line), FEM (green line), FGM (blue line) were normalized at 1022 cm^−1^. In (panel **A**), stacked spectra have been reported for the sake of clarity.

FT-IR is a useful tool in monitoring structural changes in EPS. As expected, the FT-IR spectra showed the same absorption profiles with quite similar vibrations modes (Figure 3 and Table 3). However, there were some differences, consistent with the different monosaccharide compositions and molar ratios. The intense and broad band in the range of 3600–3000 cm^−1^ region corresponds to the stretching mode of the abundant O-H or N-H moieties involved in hydrogen bond interaction. The peak at 3656 cm^−1^ detected in EPS from the FCM and FGM could be ascribed to the free OH stretching mode, which was not detected in the sample from the FEM. All the samples showed bands in the region 3000–2500 cm^−1^, which were assigned to the symmetric and asymmetric stretching vibrations of the C-H bonds in methyl groups. Only for the FCM sample was a small peak at 1730 cm^−1^ observed, which could be attributed to the C=O stretching modes of a carboxylic group. The intense bands at 1643 cm^−1^ could be ascribed to the stretching mode of the C=O of I amide or the bending of the N-H of II amide groups, ascribed to protein content. It was not possible to exclude the potential influence of water molecules trapped in the polysaccharide matrix, as absorption occurs in the same range. The amide II vibration modes are often coupled with the stretching of C-N bonds and can generate an absorption band at 1547 cm^−1^. Indeed, this band can be also ascribed to the asymmetric stretching of the carboxylates along with the symmetric ones appearing at 1406 cm^−1^. This region also contains the bending of the C-N bond, which could not be clearly distinguished from that of the carboxylates. The band at 1443 cm^−1^ can be ascribed to the asymmetric bending of C-H, whose symmetric stretching mode was visible as a shoulder of the peak at 1406 cm^−1^. The peak at 1318 cm^−1^ and that at 1244 cm^−1^ is ascribed to amide III and the C-O stretching mode. These are almost negligible in the EPS from the FEM. For all the samples, the most intense IR peak in the range of 1200–900 cm^−1^ is centered at 1024 cm^−1^ for the FCM and at 1022 cm^−1^ for the FGM and FEM. This broad band is ascribed to the C-O stretching modes coupled with those of C-C, usually attributed to the C-C-O stretching modes and is characteristic of saccharides. The increased absorption at a higher wavenumber in the 1175–1140 cm^−1^ spectral range can be attributed to a 1 → 4 glycosidic linkage, characteristic of di- and polysaccharides, as opposed to the IR spectra of monosaccharides. The clear presence of two shoulders at the higher wavenumbers 1100 cm^−1^ and 1073 cm^−1^ were detected for the FGM and FCM, confirming the presence of O-P=O, characteristic of the casein and of the C-O-C stretching modes. The band at 1073 cm^−1^ was almost negligible in the FEM sample. The weak absorptions appearing as a shoulder of the main peak at 950 cm^−1^ are attributed to O-H bending while that at 880 cm^−1^ is attributed to C-O bending, indicating the β-configuration of glycosidic bonds between monomeric units. All the peaks lower than 700 cm^−1^ are related to the skeletal signal, endocyclic and exocyclic deformation bands ascribed to components like carbohydrates, lipids, amino acids, and organic acids. Although the absorption profile is quite similar, the relative intensity between the peak at nearly 1020 cm^−1^ and that at 1643 cm^−1^, the latter being more pronounced in the EPS from the FCM than in the FGM and FEM, suggests that a higher protein content is expected compared to polysaccharides according to the following trend: FCM > FGM > FEM. Furthermore, as shown in Figure 3B, at a frequency higher than 1022 cm^−1^, more intense peaks are detected for the EPS from the FGM and FCM than from the FEM, supporting the hypothesis of a higher content of casein. 

From this framework, it can be concluded that the EPS from the FCM had a higher protein content, which could be correlated with the higher antioxidant activity compared to the EPS from the FGM and FEM. Other authors have pointed out that the antioxidant activity of EPS is affected by their components, and that high sugar and protein levels generally increase the antioxidant activity [18,61]. In addition, the relatively high protein content suggested that the EPS from the FCM are protein-bound polysaccharides [62,63]. 

### 3.8. Microstructure of Freeze-Dried EPS 

**Figure 4:**
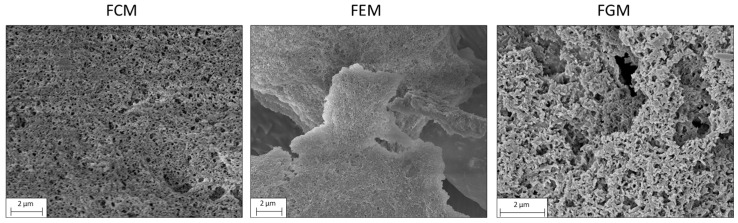
Scanning electron microscopy. The microstructure and surface morphology micrographs of EPS from FCM, FEM, and FGM were observed by SEM. Magnification operating in the range 10–25 Kx. Samples were analyzed at an accelerating voltage of 3 kV.

The study of macromolecules’ surface appearance and common physical characteristics can be accomplished with great effectiveness by using SEM. A highly organized stable three-dimensional structure that resembles a porous network was observed in the EPS samples (Figure 4). In the examined EPS samples (Figure 4), a notably organized and stable three-dimensional structure reminiscent of a porous network was discerned. Nevertheless, the variations in monosaccharide composition and functional groups, as elucidated in preceding sections, inevitably exerted an impact on the microstructures and surface morphologies of EPS purified from distinct types of milk. The EPS from the FCM had a compact structure with a wavy surface, covered with pores. The EPS from the FEM was loosely integrated and formed irregular lumps with an uneven surface and tiny pores. The EPS from the FGM showed a branched structure with spike-like expansions, resulting in a rough surface. In addition to the factors already mentioned, microstructural differences may depend on chain length and branching, which in turn are influenced by the availability of sugars or specific precursors, as well as the regulatory mechanisms that control the expression of genes involved in EPS synthesis. Interactions between the EPS and other cellular components (e.g., proteins) may also affect the degree of branching or contribute to the formation of a more complex EPS network.

## 4. Conclusions

The type of milk can affect the microbial dynamics during MK fermentation, which in turn has a significant impact on the characteristics of the fermented product. Although different types of milk are used in the production of MK, little information is available on their effect on the antioxidant properties, in particular the release of antioxidant EPS. 

Under the conditions of this study, the inoculated microbial consortium was driven differently by each type of milk (cow, ewe, and goat). These effects were evident in sugar consumption, organic acid production, free amino release, and EPS production. The amount and the composition of the secreted EPS varied depending on the type of the milk, with consequences on the structure and functional properties of the EPS. The low EPS yield in the FEM was associated with a higher lactic acid production and thus with the use of carbon sources oriented towards energy production in the context of lactic acid fermentation. Depending on the milk used as substrate, the EPS showed different monosaccharide and FT-IR profiles, microstructures, and surface morphologies. These differences had repercussions on the antiradical properties and reducing power of the EPS. In particular, the EPS extracted from the FCM had a higher antioxidant activity than the other milk types, and the antioxidant activity was concentration dependent. In addition to a different monosaccharide profile, the EPS from the FCM appeared to have a higher protein content. 

This study provides the basis for directing the production of MK towards a greater accumulation in EPS with high antioxidant potential. This can synergize with other bioactive molecules present in MK, making it ideal for the health-promoting food market. A more in-depth investigation would be desirable to define in detail the relationship between the type of milk, microbial dynamics, and the structural and functional properties of EPS.

## Figures and Tables

**Table 1:** Physicochemical parameters measured in unfermented samples of cow, ewe, and goat milk (CM, EM, GM), and in samples fermented at 25 °C for 24 h (FCM, FEM, FGM).

	TTA (mL 0.1 M NaOH per 10 mL)	pH	Viscosity (mPa·s)	L*	a*	b*	ΔE*_ab_
CM	0.2 ± 0.1 ^c^	6.58 ± 0.03 ^b^	1.11 ± 0.08 ^c^	68.17 ± 1.52 ^b^	−6.20 ± 0.18 ^a^	12.65 ± 0.88 ^b^	-
EM	0.2 ± 0.1 ^c^	6.61 ± 0.02 ^b^	1.15 ± 0.11 ^c^	78.03 ± 0.71 ^a^	−6.82 ± 0.10 ^b^	12.70 ± 0.16 ^b^	-
GM	0.2 ± 0.1 ^c^	6.73 ± 0.02 ^a^	1.13 ± 0.12 ^c^	70.12 ± 0.49 ^b^	−7.01 ± 0.14 ^b^	10.02 ± 0.17 ^c^	-
FCM	0.9 ± 0.1 ^b^	4.39 ± 0.04 ^c^	1.59 ± 0.15 ^ab^	76.97 ± 0.51 ^a^	−6.85 ± 0.11 ^b^	17.50 ± 0.14 ^a^	10.07 ± 2.22 ^a^
FEM	2.1 ± 0.3 ^a^	4.06 ± 0.05 ^e^	1.37 ± 0.17 ^bc^	76.96 ± 0.42 ^a^	−6.11 ± 0.18 ^a^	11.96 ± 0.30 ^b^	1.48 ± 0.34 ^b^
FGM	1.0 ± 0.2 ^b^	4.19 ± 0.07 ^d^	1.62 ± 0.13 ^ab^	69.87 ± 1.07 ^b^	−6.30 ± 0.14 ^a^	10.57 ± 0.17 ^c^	1.05 ± 0.13 ^b^

^a–e^ Data were subjected to one-way ANOVA followed by Tukey’s procedure at *p* < 0.05. Means within the columns with different superscript letters differ significantly (*p* < 0.05).

**Table 2:** Concentrations (g L^−1^) of sugars, organic acids, and ethanol in unfermented samples of cow, ewe, and goat milk (CM, EM, GM), and in samples fermented at 25 °C for 24 h (FCM, FEM, FGM).

	Lactose	Glucose	Galactose	Xylitol	Lactic Acid	Acetic Acid	Ethanol
CM	49.94 ± 1.77 ^a^	n.d.	n.d.	n.d.	n.d.	n.d.	n.d.
EM	45.63 ± 0.39 ^b^	n.d.	n.d.	n.d.	n.d.	n.d.	n.d.
GM	42.74 ± 0.38 ^c^	n.d.	n.d.	n.d.	n.d.	n.d.	n.d.
FCM	33.20 ± 3.47 ^d^	n.d.	0.80 ± 0.05	0.09 ± 0.01 ^b^	15.21 ± 0.91 ^b^	0.35 ± 0.02 ^b^	n.d.
FEM	23.04 ± 0.39 ^e^	0.88 ± 0.02	0.88 ± 0.02	0.23 ± 0.01 ^a^	21.80 ± 0.51 ^a^	0.43 ± 0.01 ^a^	n.d.
FGM	24.19 ± 1.87 ^e^	n.d.	0.89 ± 0.05	0.08 ± 0.01 ^b^	14.06 ± 0.98 ^b^	0.29 ± 0.01 ^c^	n.d.

^a–e^ Data were subjected to one-way ANOVA followed by Tukey’s procedure at *p* < 0.05. Means within the columns with different superscript letters differ significantly (*p* < 0.05). n.d., not detected.

**Table 3:** FT-IR spectra in ATR mode. Table reporting the absorption wavenumber for EPS from FCM, FEM, and FGM.

	FCM (cm^−1^)	FEM (cm^−1^)	FGM (cm^−1^)
ν_s_ OH monomer H-bonded	3660		3660
ν_s_ OH dimer H-bond	3278	3278	3278
ν_s_ CH_3_	2986	2986	2986
ν_s_ CH_2_	2970	2970	2970
ν_as_ CH_3_	2922	2922	2922
ν_as_ CH_2_	2901	2901	2901
C=O carboxylic group	1730		
δ H_2_O/ν C=O amide I and δ N-H amide II	1643	1643	1643
ν_as_ COO^−^/δ N-H amide II coupled with ν C-N	1547	1547	1547
δ_s_ CH_2_	1454	1454	1454
ν_s_ COO^−^/ν C-N	1406	1406	1406
amide III and C-O stretching mode	1318 1244	1318 1244	1318 1244
C-C-O stretching modes/O-P=O modes	1100, 1080, 1024	1100, 1022	1100, 1080, 1022
δOH	950	950	950

## Data Availability

Data are contained within the article.

## References

[1] Farag M.A., Jomaa S.A., Abd El-Wahed A., El-Seedi H.R. (2020). The many faces of kefir fermented dairy products: Quality characteristics, flavour chemistry, nutritional value, health benefits, and safety. Nutrients.

[2] Izquierdo-González J.J., Amil-Ruiz F., Zazzu S., Sánchez-Lucas R., Fuentes-Almagro C.A., Rodríguez-Ortega M.J. (2019). Proteomic analysis of goat milk kefir: Profiling the fermentation-time dependent protein digestion and identification of potential peptides with biological activity. Food Chem..

[3] Wang H., Sun X., Song X., Guo M. (2021). Effects of kefir grains from different origins on proteolysis and volatile profile of goat milk kefir. Food Chem..

[4] El S.N., Karakaya S., Simsek S., Dupont D., Menfaatli E., Eker A.T. (2015). In vitro digestibility of goat milk and kefir with a new standardised static digestion method (INFOGEST cost action) and bioactivities of the resultant peptides. Food Funct..

[5] Dalabasmaz S., De la Torre E.P., Gensberger-Reigl S., Pischetsrieder M., Rodríguez-Ortega M.J. (2023). Identification of Potential Bioactive Peptides in Sheep Milk Kefir through Peptidomic Analysis at Different Fermentation Times. Foods.

[6] Ferreira I.M.P.L.V.O., Pinho O., Monteiro D., Faria S., Cruz S., Perreira A., Tavares P. (2010). Effect of kefir grains on proteolysis of major milk proteins. J. Dairy Sci..

[7] Gentry B., Cazón P., O’Brien K. (2023). A comprehensive review of the production, beneficial properties, and applications of kefiran, the kefir grain exopolysaccharide. Int. Dairy J..

[8] De Carvalho A.P.A., Conte-Junior C.A. (2021). Food-derived biopolymer kefiran composites, nanocomposites and nanofibers: Emerging alternatives to food packaging and potentials in nanomedicine. Trends Food Sci. Technol..

[9] Bengoa A.A., Dueñas M.T., Prieto A., Garrote G.L., Abraham A.G. (2023). Exopolysaccharide-producing *Lacticaseibacillus paracasei* strains isolated from kefir as starter for functional dairy products. Front. Microbiol..

[10] Jiang S.J., Qian F., Ren X.H., Mu G.Q. (2013). Studies on the preliminary characterization of a novel exopolysaccharide produced by Streptococcus thermophilus strain from Tibetan kefir grain. Adv. Mat. Res..

[11] Wang L., Gu Y., Zheng X., Zhang Y., Deng K., Wu T., Cheng H. (2021). Analysis of physicochemical properties of exopolysaccharide from *Leuconostoc mesenteroides* strain XR1 and its application in fermented milk. LWT.

[12] Chen Z., Shi J., Yang X., Nan B., Liu Y., Wang Z. (2015). Chemical and physical characteristics and antioxidant activities of the exopolysaccharide produced by Tibetan kefir grains during milk fermentation. Int. Dairy J..

[13] Wang W., Ju Y., Liu N., Shi S., Hao L. (2023). Structural characteristics of microbial exopolysaccharides in association with their biological activities: A review. Chem. Biol. Technol. Agric..

[14] Adebayo-Tayo B., Ishola R., Oyewunmi T. (2018). Characterization, antioxidant and immunomodulatory potential on exopolysaccharide produced by wild type and mutant *Weissella confusa* strains. Biotechnol. Rep..

[15] Zhao Z.Y., Zhang Q., Li Y.F., Dong L.L., Liu S.L. (2015). Optimization of ultrasound extraction of *Alisma orientalis* polysaccharides by response surface methodology and their antioxidant activities. Carbohydr. Polym..

[16] Jahanbin K., Gohari A.R., Moini S., Emam-Djomeh Z., Masi P. (2011). Isolation, structural characterization and antioxidant activity of a new water-soluble polysaccharide from *Acanthophyllum bracteatum* roots. Int. J. Biol. Macromol..

[17] Zhang Z., Lv G., He W., Shi L., Pan H., Fan L. (2013). Effects of extraction methods on the antioxidant activities of polysaccharides obtained from *Flammulina velutipes*. Carbohydr. Polym..

[18] Hasheminya S.M., Dehghannya J. (2020). Novel ultrasound-assisted extraction of kefiran biomaterial, a prebiotic exopolysaccharide, and investigation of its physicochemical, antioxidant and antimicrobial properties. Mater. Chem. Phys..

[19] Andrew M., Jayaraman G. (2020). Structural features of microbial exopolysaccharides in relation to their antioxidant activity. Carbohydr. Res..

[20] Buran İ., Akal C., Ozturkoglu-Budak S., Yetisemiyen A. (2021). Rheological, sensorial and volatile profiles of synbiotic kefirs produced from cow and goat milk containing varied probiotics in combination with fructooligosaccharide. LWT.

[21] Al-Kaisy Q.H., Al-Saadi J.S., Al-Rikabi A.K.J., Altemimi A.B., Hesarinejad M.A., Abedelmaksoud T.G. (2023). Exploring the health benefits and functional properties of goat milk proteins. Food Sci. Nutr..

[22] Flis Z., Molik E. (2021). Importance of Bioactive Substances in Sheep’s Milk in Human Health. Int. J. Mol. Sci..

[23] Prado M.R., Blandón L.M., Vandenberghe L.P., Rodrigues C., Castro G.R., Thomaz-Soccol V., Soccol C.R. (2015). Milk kefir: Composition, microbial cultures, biological activities, and related products. Front. Microbiol..

[24] Zhong Z., Hou Q., Kwok L., Yu Z., Zheng Y., Sun Z., Zhang H. (2016). Bacterial microbiota compositions of naturally fermented milk are shaped by both geographic origin and sample type. J. Dairy Sci..

[25] Kim Y.H., Kim J.U., Oh S.J., Kim Y.J., Kim M.H., Kim S.H. (2008). Technical optimization of culture conditions for the production of exopolysaccharide (EPS) by *Lactobacillus rhamnosus* ATCC 9595. Food Sci. Biotechnol..

[26] Wang X., Li W., Xu M., Tian J., Li W. (2021). The microbial diversity and biofilm-forming characteristic of two traditional Tibetan kefir grains. Foods.

[27] Luang-In V., Deeseenthum S. (2016). Exopolysaccharide-producing isolates from Thai milk kefir and their antioxidant activities. LWT.

[28] M’hir S., Rtibi K., Ayed L., Hamdi M., Marzouki L., Sebai H. (2019). Evaluation de l’effet protecteur du lait de chèvre fermenté par le kefir et enrichi à la caroube contre l’ulcère gastrique induit par l’étahnol chez le rat. Int. J. Adv. Res..

[29] Kim D.-H., Jeong D., Song K.-Y., Seo K.-H. (2018). Comparison of traditional and backslopping methods for kefir fermentation based on physicochemical and microbiological characteristics. LWT.

[30] Alves E., Ntungwe E.N., Gregório J., Rodrigues L.M., Pereira-Leite C., Caleja C., Pereira E., Barros L., Aguilar-Vilas M.V., Rosado C. (2021). Characterization of kefir produced in household conditions: Physicochemical and nutritional profile, and storage stability. Foods..

[31] Tlais A.Z.A., Kanwal S., Filannino P., Albiac M.A., Gobbetti M., Di Cagno R. (2022). Effect of sequential or ternary starters-assisted fermentation on the phenolic and glucosinolate profiles of sauerkraut in comparison with spontaneous fermentation. Food Res. Int..

[32] Verni M., Dingeo C., Rizzello C.G., Pontonio E. (2021). Lactic Acid Bacteria Fermentation and Endopeptidase Treatment Improve the Functional and Nutritional Features of *Arthrospira platensis*. Front. Microbiol..

[33] Bouallegue A., Casillo A., Chaari F., La Gatta A., Lanzetta R., Corsaro M.M., Bachoual R., Ellouz-Chaabouni S. (2020). Levan from a new isolated *Bacillus subtilis* AF17: Purification, structural analysis and antioxidant activities. Int. J. Biol. Macromol..

[34] Groth S., Budke C., Neugart S., Ackermann S., Kappenstein F.S., Daum D., Rohn S. (2020). Influence of a selenium biofortification on antioxidant properties and phenolic compounds of apples (*Malus domestica*). Antioxidants.

[35] Di Cagno R., De Angelis M., Limitone A., Minervini F., Carnevali P., Corsetti A., Gäenzle M., Ciati R., Gobbetti M. (2006). Glucan and fructan production by sourdough *Weissella cibaria* and *Lactobacillus plantarum*. J. Agric. Food Chem..

[36] M’hir S. (2023). (National Institute of Applied Sciences and Technology (INSAT), Tunis, Tunisia).

[37] Öner Z., Karahan A.G., Çakmakçı M.L. (2015). Effects of different milk types and starter cultures on kefir. GIDA.

[38] Guangsen T., Xiang L., Jiahu G. (2021). Microbial diversity and volatile metabolites of kefir prepared by different milk types. CYTA J. Food.

[39] Horne D.S. (1998). Casein interactions: Casting light on the black boxes, the structure in dairy products. Int. Dairy J..

[40] Tamime A.Y., Robinson R.K. (2007). Tamime and Robinson’s Yoghurt—Science and Technology.

[41] Tamime A.Y., Robinson R.K., Tamime A.Y., Robinson R.K. (1999). Background to manufacturing practice. Yoghurt: Science and Technology.

[42] Yang T., Wu K., Wang F., Liang X., Liu Q., Li G., Li Q. (2014). Effect of exopolysaccharides from lactic acid bacteria on the texture and microstructure of buffalo yoghurt. Int. Dairy J..

[43] Amatayakul T., Halmos A.L., Sherkat F., Shah N.P. (2006). Physical characteristics of yoghurts made using exopolysaccharide-producing starter cultures and varying casein to whey protein ratios. Int. Dairy J..

[44] Ruas-Madiedo P., Tuinier R., Kanning M., Zoon P. (2002). Role of exopolysaccharides produced by *Lactococcus lactis* subsp. cremoris on the viscosity of fermented milks. Int. Dairy J..

[45] Koroleva N.S. (1975). Starters for fermented milks. Section 4, Kefir and Kumys starters. Bulletion IDF.

[46] Saygili D., Döner D., İçier F., Karagözlü C. (2021). Rheological properties and microbiological characteristics of kefir produced from different milk types. Food Sci. Technol..

[47] Rizzello C.G., Portincasa P., Montemurro M., Di Palo D.M., Lorusso M.P., De Angelis M., Bonfrate L., Genot B., Gobbetti M. (2019). Sourdough Fermented Breads are More Digestible than Those Started with Baker’s Yeast Alone: An In Vivo Challenge Dissecting Distinct Gastrointestinal Responses. Nutrients.

[48] Gamba R.R., Yamamoto S., Abdel-Hamid M., Sasaki T., Michihata T., Koyanagi T., Enomoto T. (2020). Chemical, Microbiological, and Functional Characterization of Kefir Produced from Cow’s Milk and Soy Milk. Int. J. Microbiol..

[49] Collar C., Mascarós A.F., Barber C.B. (1992). Amino Acid Metabolism by Yeasts and Lactic Acid Bacteria during Bread Dough Fermentation. J. Food Sci..

[50] Grønnevik H., Falstad M., Narvhus J.A. (2011). Microbiological and chemical properties of Norwegian kefir during storage. Int. Dairy J..

[51] Ziarno M., Hasalliu R., Cwalina A. (2021). Effect of the Addition of Milk Protein Preparations on Selected Quality Parameters and Nutritional Characteristics of Kefir. Appl. Sci..

[52] Rimada P.S., Abraham A.G. (2021). Polysaccharide production by kefir grains during whey fermentation. J. Dairy Res..

[53] Wang Y., Ahmed Z., Feng W., Li C., Song S. (2008). Physicochemical properties of exopolysaccharide produced by *Lactobacillus kefiranofaciens* ZW3 isolated from Tibet kefir. Int. J. Biol. Macromol..

[54] Radhouani H., Gonçalves C., Maia F.R., Oliveira J.M., Reis R.L. (2018). Biological performance of a promising Kefiran-biopolymer with potential in regenerative medicine applications: A comparative study with hyaluronic acid. J. Mater. Sci. Mater. Med..

[55] Blandón L.M., Noseda M.D., Islan G.A., Castro G.R., De Melo Pereira G.V., Thomaz-Soccol V., Soccol C.R. (2018). Optimization of culture conditions for kefiran production in whey: The structural and biocidal properties of the resulting polysaccharide. Bioact. Carbohydr. Diet. Fibre.

[56] Xiao L., Xu D., Tang N., Rui X., Zhang Q., Chen X., Li W. (2021). Biosynthesis of exopolysaccharide and structural characterization by *Lacticaseibacillus paracasei* ZY-1 isolated from Tibetan kefir. Food Chem..

[57] Koçak Ç., Demiralay E.Ç., Özarslan S., Aydoğdu N.S., Taş T.K. (2021). Determination of monosaccharide composition of kefiran using HPLC. Mljekarstvo.

[58] Ahmed Z., Wang Y., Anjum N., Ahmad A., Khan S.T. (2013). Characterization of exopolysaccharide produced by *Lactobacillus kefiranofaciens* ZW3 isolated from Tibet kefir–Part II. Food Hydrocoll..

[59] Ahmed Z., Wang Y., Anjum N., Ahmad H., Ahmad A., Raza M. (2013). Characterization of new exopolysaccharides produced by coculturing of *L. kefiranofaciens* with yoghurt strains. Int. J. Biol. Macromol..

[60] Zannini E., Lynch K.M., Nyhan L., Sahin A.W., O’Riordan P., Luk D., Arendt E.K. (2022). Influence of substrate on the fermentation characteristics and culture-dependent microbial composition of water kefir. Fermentation.

[61] Li W., Ji J., Chen X., Jiang M., Rui X., Dong M. (2014). Structural elucidation and antioxidant activities of exopolysaccharides from *Lactobacillus helveticus* MB2-1. Carbohydr. Polym..

[62] Qiao D., Ke C., Hu B., Luo J., Ye H., Sun Y., Zeng X. (2009). Antioxidant activities of polysaccharides from *Hyriopsis cumingii*. Carbohydr. Polym..

[63] Qiao D., Liu J., Ke C., Sun Y., Ye H., Zeng X. (2010). Structural characterization of polysaccharides from *Hyriopsis cumingii*. Carbohydr. Polym..

